# Comparative Analysis of the *Flavobacterium columnare* Genomovar I and II Genomes

**DOI:** 10.3389/fmicb.2017.01375

**Published:** 2017-07-25

**Authors:** Salih Kumru, Hasan C. Tekedar, Nagihan Gulsoy, Geoffrey C. Waldbieser, Mark L. Lawrence, Attila Karsi

**Affiliations:** ^1^Department of Basic Sciences, College of Veterinary Medicine, Mississippi State University Mississippi State, MS, United States; ^2^Department of Biology, Faculty of Art and Sciences, Marmara University Istanbul, Turkey; ^3^Warmwater Aquaculture Research Unit, United States Agricultural Research Service, Stoneville MS, United States

**Keywords:** *Flavobacterium columnare*, genomovars, comparative genomics, catfish, transposon mutagenesis, virulence, fish health

## Abstract

Columnaris disease caused by Gram-negative rod *Flavobacterium columnare* is one of the most common diseases of catfish. *F. columnare* is also a common problem in other cultured fish species worldwide. *F. columnare* has three major genomovars; we have sequenced a representative strain from genomovar I (ATCC 49512, which is avirulent in catfish) and genomovar II (94-081, which is highly pathogenic in catfish). Here, we present a comparative analysis of the two genomes. Interestingly, *F. columnare* ATCC 49512 and 94-081 meet criteria to be considered different species based on the Average Nucleotide Identity (90.71% similar) and DNA–DNA Hybridization (42.6% similar). Genome alignment indicated the two genomes have a large number of rearrangements. However, function-based comparative genomics analysis indicated that the two strains have similar functional capabilities with 2,263 conserved orthologous clusters; strain ATCC 49512 has 290 unique orthologous clusters while strain 94-081 has 391. Both strains carry type I secretion system, type VI secretion system, and type IX secretion system. The two genomes also have similar CRISPR capacities. The *F. columnare* strain ATCC 49512 genome contains a higher number of insertion sequence families and phage regions, while the *F. columnare* strain 94-081 genome has more genomic islands and more regulatory gene capacity. Transposon mutagenesis using Tn4351 in pathogenic strain 94-081 yielded six mutants, and experimental infections of fish showed hemolysin and glycine cleavage protein mutants had 15 and 10% mortalities, respectively, while the wild-type strain caused 100% mortalities. Our comparative and mutational analysis yielded important information on classification of genomovars I and II *F. columnare* as well as potential virulence genes in *F. columnare* strain 94-081.

## Introduction

Channel catfish is the most important aquaculture commodity in Mississippi and the largest aquaculture industry in the United States, but it is impacted negatively by columnaris disease caused by *Flavobacterium columnare*. In the United States, the disease causes losses up to $30 million annually (Shoemaker et al., 2011; Declercq et al., 2013). Columnaris outbreaks may result in high mortality, especially during spring and autumn, and are associated with stressful conditions, including high temperatures, elevated organic loads, crowded ponds, and excessive handling (Wakabayashi, 1991). *F. columnare* causes acute to chronic infections and affects gills, skin, and fins; in particular, dorsal fin and surrounding skin are often affected, causing “saddleback” lesions. When the disease is acute or subacute, yellowish areas of necrotic tissue can be seen in the gills, resulting in severe gill pathology (Decostere, 2002).

*F. columnare* was isolated by Davis in 1922 (Davis, 1922) and named *Bacillus columnaris*. After several reclassifications, a final name was assigned in 1996 (Bernardet et al., 1996). *F. columnare* is a Gram-negative, long rod in the family *Flavobacteriaceae* (Davis, 1922; Bernardet et al., 1996). *F. columnare* demonstrates varying colony morphologies and genetic heterogeneity as well as significant variation in virulence in different fish species. *F. columnare* strains are assigned into one of three genomovar groups by using 16S rDNA restriction fragment length polymorphism analysis (Triyanto and Wakabayash, 1999).

*Flavobacterium columnare* ATCC 49512, belonging to genomovar group I (Michel et al., 2002), was isolated from a brown trout skin lesion in France in 1987 (Bernardet, 1989), and it is not virulent to catfish (Soto et al., 2008). *F. columnare* genomovar II is the most virulent genomovar group in warm water fish species, including catfish (Triyanto and Wakabayash, 1999; Arias et al., 2004; Darwish and Ismaiel, 2005; Olivares-Fuster et al., 2007; Shoemaker et al., 2008; Bullard et al., 2013). However, genomovar I isolates are more virulent than genomovar II isolates in rainbow trout challenges (Evenhuis and LaFrentz, 2016). *F. columnare* strain 94-081 is in genomovar II and is highly virulent in catfish (Soto et al., 2008; Staroscik et al., 2008; Lawrence et al., 2012).

*Flavobacterium johnsoniae* UW101 genome was the first completed *Flavobacterium* genome reported (accession # CP000685) (McBride et al., 2009). Later, the genome of *Flavobacterium psychrophilum* JIP02/86, the causative agent of cold water disease in salmonid fish, was reported (accession # 511344733) (Duchaud et al., 2007). *Flavobacterium branchiophilum* FL-15 genome was finished in 2011 (accession # FQ859183) (Touchon et al., 2011), and our group sequenced the complete genome of *F. columnare* genomovar I strain ATCC 49512 (accession # CP003222.2) (Tekedar et al., 2012). *F. columnare* genomovar II strain 94-081 was recently sequenced and completed by our group (accession # CP013992.1) (Kumru et al., 2016).

Comparative analysis of complete genomes provides an excellent opportunity to determine unique genomic features. Availability of non-virulent and virulent strains belonging to genomovars I and II should reveal potential virulence factors and mechanisms causing columnaris disease. Here, we report the first comparative analysis of *F. columnare* strain ATCC 49512 (genomovar I) and strain 94-081 (genomovar II) genomes. Moreover, random transposon mutagenesis in strain 94-081 was conducted to identify potential virulence genes. We expect that the new knowledge gained from this study will clarify the systematic classification of genomovar I and II *F. columnare* and help elucidate the pathogenesis of columnaris disease.

## Materials and Methods

### Bacterial Species and Growth Conditions

Bacterial strains and plasmids used in this study are listed in **Table 1**. *F. columnare* strain 94-081 was grown at 30°C in FCGM agar plate and FCGM broth with shaking at 200 rpm (Farmer, 2004). *E. coli* strains were cultured at 37°C in the Luria-Bertani (LB) medium and LB broth with shaking at 200 rpm.

**Table 1:** Bacterial strains and plasmids.

Strains	Reference
*Flavobacterium columnare* 94-081	Soto et al., 2008
*Escherichia coli* S17-1 λ*pir*	Simon et al., 1983
**Plasmids**	
pEP4351 (Tn4351 transposon)	

### Genomic DNA Extraction and Sequencing

For genomic DNA extraction from *F. columnare* strain 94-081 and transposon mutants, the CTAB/NaCI protocol (Murray and Thompson, 1980; Wilson, 2001) was used with some modifications. The 1.5 ml of *F. columnare* strain 94-081 culture was pelleted by centrifugation for 2 m at 12,000 rpm. The supernatant was discarded, the pellet was resuspended in 567 μl of TE buffer, and 20 μl of 10 mg/ml RNAse A was added. After 1 h incubation at 37°C, 40 μl of 10% SDS was added. After gentle mixing, 6 μl of 10 mg/ml proteinase K was added. The sample was incubated for 1 h at 56°C, after which 100 μl of 5 M NaCl was added with thorough mixing. The 80 μl of CTAB/NaCl solution was added, and the sample was incubated at 65°C for 10 m. An equal volume of chloroform/isoamyl alcohol was added followed by centrifugation for 5 m at 14,000 rpm. The aqueous phase was transferred to a fresh tube. The 0.7 ml of phenol/chloroform/isoamyl alcohol was added, followed by gentle mixing and centrifugation for 5 m at 14,000 rpm. The aqueous phase was transferred to a fresh tube. The 0.6 ml of isopropanol was added, and the centrifugation was repeated. Finally, after discarding the supernatant, precipitated DNA was washed by adding 1 ml of 70% ethanol, and centrifugation was conducted for 5 m at 12,000 rpm. The supernatant was removed, and the pellet was dried briefly at room temperature and resuspended in 100 μl of TE buffer. Genomic DNA sequencing was conducted as described (Kumru et al., 2016).

### Genome Annotation and Comparative Analysis

*F. columnare* strain ATCC 49512 and strain 94-081 genome sequence accession numbers are CP003222 and CP013992, respectively. For annotation and coding protein comparison, we applied RAST (Overbeek et al., 2014) and NCBI PGAAP (Angiuoli et al., 2008) annotation tools. For RAST annotation, nucleotide files were uploaded to RAST by default features (RAST annotation scheme: classic RAST, gene caller: RAST, FIGfam version: Release70, automatically fix errors, fix frameshifts, build metabolic model, backfill gaps, turn on debug, and disable replication: yes, verbose level: 0). Mauve multiple genome alignment tool (Darling et al., 2010) was used with default settings for comparison of genomes. To evaluate general genetic similarity, two-way Average Nucleotide Identity (ANI) (Goris et al., 2007) and DNA–DNA hybridization (DDH) assessments (Meier-Kolthoff et al., 2013) were conducted using recommended default settings. A phylogenetic tree using the complete genomes of 22 *Flavobacterium* genus members was built from their core genomes (as of 04/2017). The core genome gene sets were aligned one by one using MUSCLE (Edgar, 2004) followed by concatenation of the alignments. Alignment results were used to compute Kimura distance matrix, which was used as input for the Neighbor-Joining algorithm as implemented in the PHYLIP package (Felsenstein, 1989).

To predict bacterial protein secretion systems, analysis with MacSyFinder was performed using default features (unordered replicon, circular, all available systems, maximal *E*-value 1.0, maximal independent *E*-value 0.001, minimal profile coverage 0.5) (Abby et al., 2014). CRISPRFinder was used to detect the clustered regularly interspaced short palindromic repeats (CRISPRs) and specific families of tandem repeats (Grissa et al., 2007). To determine phage elements, PHASTER was used (Arndt et al., 2016). To identify genomic islands (GIs), IslandViewer3^1^, which integrates three different identification approaches (IslandPick, SIGI-HMM, IslandPath-DIMOB), was applied (Hsiao et al., 2003; Waack et al., 2006; Langille et al., 2008; Dhillon et al., 2015). To determine insertion sequences (ISs), ISsaga^2^ was used (Varani et al., 2011). For identification and analysis of signal transduction regulatory proteins, P2RP^3^ was used (Barakat et al., 2013). Orthologous genes analysis was performed using OrthoVenn^4^ with default features (*E*-value 1*e* - 5, inflation value 1.5) (Wang et al., 2015). Virulence factors were determined by downloading MvirDB^5^ (Zhou et al., 2007) and constructing a searchable database in Genomics Workbench 6.5.1 with a cutoff *E*-value of 10^-10^.

### Random Transposon Mutagenesis

Random transposon mutagenesis in *F. columnare* 94-081 was performed as described previously with some modifications (McBride and Kempf, 1996; Karlyshev et al., 2000; Karsi et al., 2009). Briefly, *F. columnare* 94-081 and donor *E. coli* S17-1 λ*pir* strain with pEP4351 were grown to mid-log phase. Then bacteria were harvested by centrifugation at 12,000 rpm for 2 m. *F. columnare* and donor *E. coli* were mixed at 1:2 or 1:4 ratios and spotted onto a filter paper placed on FCGM agar. Following overnight incubation at 30°C, bacteria were removed from the filter paper by washing with FGCM broth, and serial dilutions were prepared. Diluted bacteria were spread on FCGM agar containing erythromycin (10 μg/ml) and colistin (50 μg/ml). During development of the method, lower concentrations of erythromycin were used (1 μg/ml and 5 μg/ml), but some background non-mutant colonies occurred. At 10 μg/ml, erythromycin-resistant *F. columnare* colonies appeared after 2 days of incubation at 30°C. Tn4351 insertion sites were identified using a single-primer PCR method (Karlyshev et al., 2000; Karsi et al., 2009). Tn4351 ends were amplified using specific left (atcaggcagtatatcccλgg) or right (attgcgctttatctccctgtaa) primers, and amplicons were sequenced using nested left (atcgacctcgtλagacttgg) or right (ggacggacaattλgcλga) primers. Sequences were searched using BLASTX for identification of genes with Tn4351 insertion.

### Fish Virulence

This study was conducted by a protocol approved by the Mississippi State University Institutional Animal Care and Use Committee. Virulence of mutants was compared to parent strain 94-081 in channel catfish as described (Karsi et al., 2009). Briefly, 640 specific-pathogen-free (SPF) channel catfish fingerlings (14.65 ± 1.43 cm, 24.75 ± 6.50 g) were stocked into 32 40-L flow-through tanks (flow rate: 1 L/m) at a rate of 20 fish/tank and acclimated for 1 week. Chlorine, dissolved oxygen, and temperature were monitored daily. Four tanks were randomly assigned to each *F. columnare* mutant (total six mutants), *F. columnare* strain 94-081, and negative control. Immersion challenge was applied by lowering the water in each tank to 10-L and by adding 100 ml overnight culture (adjusted to equivalent bacterial concentration using optical density at 600 nm). Infection dose (average 1.93 × 10^7^ CFU/ml water) was determined by serial dilution and plate counting. Negative control treatment tanks were exposed to an equal volume of sterile FCGM. After 5 h of incubation under aerated conditions, water flow was restored to each tank, and mortalities were recorded daily for a total of 8 days.

## Results

### Genome Features

The circular genome size of *F. columnare* strain ATCC 49512 is 3,162,432 bp (G+C 31.5%), which has 2,632 predicted protein coding genes, 1,121 of which are hypothetical proteins. The genome includes 5 ribosomal RNA operons (5S, 16S, and 23S) (two tandem and three scattered operons), and 74 tRNAs. RAST annotation showed 318 subsystems in the *F. columnare* strain ATCC 49512 genome (**Table 2**).

**Table 2:** Genome features of *F. columnare* ATCC 49512 and 94-081.

Features/Strain	ATCC 49512	94-081
Status	Completed	Completed
Source	Brown Trout	Diseased Catfish
Country of isolation	France	United States
Date of isolation	1987	1994
Genome size	3,162,432	3,321,600
G+C content (%)	31.5	30.8
Number of total protein	2,632	2,779
Number of total genes	2,772	2,897
Complete rRNAs operons	5	4
Number of total rRNA	15	12
Number of total tRNA	74	74
Number of other RNA	1	3
Number of pseudogene genes	50	29
Number of hypothetical protein	1,121	1,158
Number of subsystems	318	324
Compared function based unique genes	10	34
Sequence-based unique genes	393	412

The circular genome size of *F. columnare* strain 94-081 is 3,321,600 bp. The chromosome is predicted to contain 2,779 protein-coding genes, 1,158 of which encode hypothetical proteins. The genome has 4 ribosomal RNA (5S, 16S, and 23S) operons (two of which are located in tandem), 74 tRNAs, and 3 non-coding RNAs. RAST annotation showed 324 subsystems in the *F. columnare* strain 94-081 genome (**Table 2**).

**FIGURE 1:**
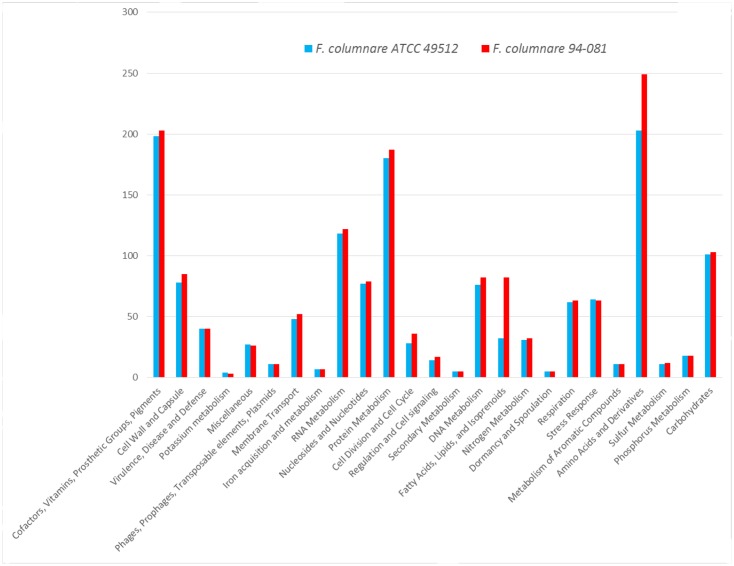
*F. columnare* ATCC 49512 and *F. columnare* 94-081 genome subsystems by RAST annotation.

Gene function analysis by RAST showed that strain ATCC 49512 contains 393 unique coding genes, and strain 94-081 has 412 unique coding genes (**Figure 1**). In particular, genes unique to strain 94-081 encode biosynthetic enzymes for amino acids and derivatives, cofactor synthetic enzymes, cell wall synthesis enzymes, DNA metabolism proteins, membrane transport proteins, cell division proteins, regulation and cell signaling proteins, and carbohydrate metabolism enzymes.

### Phylogeny Analysis

**FIGURE 2:**
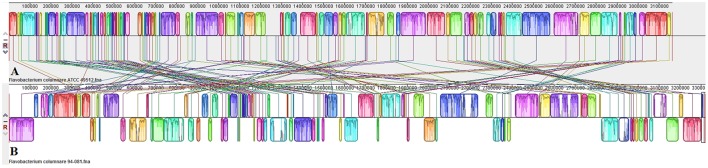
Comparison of *F. columnare* ATCC 49512 **(A)** and *F. columnare* 94-081 **(B)** genomes using MAUVE genome alignment.

**FIGURE 3:**
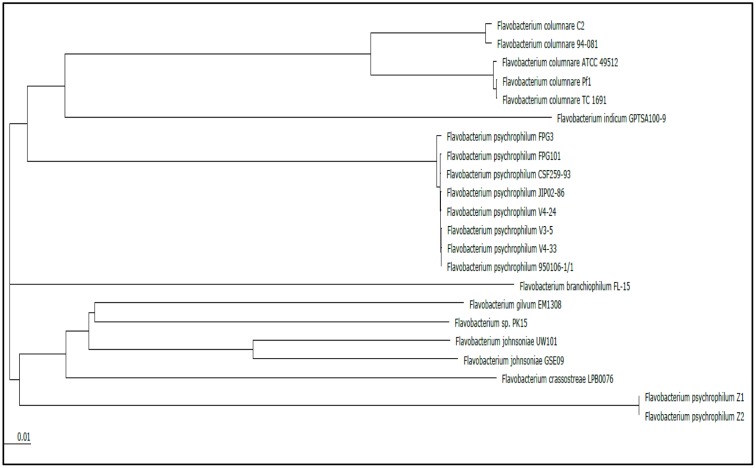
Phylogenetic tree based on comparison of the core genomes from all the current complete genomes in the *Flavobacterium* genus.

Genome alignment by Mauve showed that although the *F. columnare* ATCC 49512 and *F. columnare* 94-081 genomes have local synteny of gene clusters, extensive genomic rearrangements are present (**Figure 2**). Two-way ANI is 90.71%, and DDH approximation is 42.60%. A phylogenetic tree was built based on the core genomes of 22 *Flavobacterium* strains with complete genomes (**Figure 3**). The core *Flavobacterium* genome consisted of 1,025 genes, which encompassed 1,073,527 bp per genome (23,617,594 bp total for all 22 genomes).

### Secretion Systems

The *F. columnare* ATCC 49512 genome has a type I secretion system (T1SS), a partial type II secretion system (T2SS), a partial type III secretion system (T3SS), type IV pilus (T4P; homologous to components of the T2SS), type IV secretion system (T4SS) elements, type VI secretion system subtype 3 (T6SS^iii^), and type IX secretion system (T9SS). The *F. columnare* strain 94-081 genome contains a T1SS, T3SS, T4P, T4SS, T6SS^iii^, and T9SS as a complete operon or partial elements (**Table 3**).

**Table 3:** Predicted protein secretion systems in *F. columnare* ATCC 49512 and 94-081.

Secretion systems	Role in the system^∗^	Genes	Number of genes available in *F. columnare* ATCC 49512	Number of genes available in *F. columnare* 94-081
T1SS	Mandatory	T1SS-abc	6	6
		T1SS-mfp	3	3
		T1SS-omf	4	3
T2SS	Mandatory	T2SS-gspJ	1	0
T3SS	Mandatory	T3SS-sctN	2	2
T4P	Mandatory	T4P-pilB	1	1
		T4P-pilC	1	1
		T4P-pilM	2	1
		T4P-pilQ	1	1
T4SS	Mandatory	T4SS-t4cp2	1	0
	Accessory	T4SS-G-tfc18	0	1
		T4SS-I-traP	0	1
T6SS^i^	Mandatory	T6SSi-tssH	2	2
T6SS^iii^	Mandatory	T6SSiii-tssB	1	1
		T6SSiii-tssC	1	1
		T6SSiii-tssD	4	5
		T6SSiii-tssE	1	1
		T6SSiii-tssF	1	1
		T6SSiii-tssG	1	1
		T6SSiii-tssH	1	1
		T6SSiii-tssI	3	6
		T6SSiii-tssK	1	1
		T6SSiii-tssN	1	1
		T6SSiii-tssP	1	1
T9SS	Mandatory	T9SS-gldK	1	1
		T9SS-gldL	1	1
		T9SS-gldM	1	1
		T9SS-gldN	1	1
		T9SS-sprA	1	1
		T9SS-sprE	1	1
		T9SS-sprT	1	1
	Accessory	T9SS-porQ	2	2
		T9SS-porU	1	1
		T9SS-porV	1	1
		T9SS-gldJ	1	1

*^∗^Mandatory components are essential for system function. Accessory components are not essential for the system*.

### CRISPR-Cas System

Results of the CRISPRs analysis are summarized in **Table 4**. The *F. columnare* ATCC 49512 genome contains two CRISPR loci; one has 43 spacers, and the other has 8 spacers. Direct repeat (DR) lengths are 36 bp. The first CRISPR locus is located near a pseudogene (possibly CRISPR-associated *Cas*2 genes), and the second CRISPR locus is between genes encoding hypothetical proteins. While the CRISPR-associated *Cas*1 and possible *Cas2* genes are adjacent in the genome, the *Cas*9 gene is at a different location. A questionable third CRISPR is located near the CRISPR-associated *Cas*9 gene. The strain ATCC 49512 genome also encodes *Cas*9 type II and *Cas*1 type II proteins.

**Table 4:** CRISPRs in *F. columnare* ATCC 49512 and 94-081.

	CRISPR begin	CRISPR end	No. of spacers	DR length	CRISPR length (bp)
***Flavobacterium columnare* 94-081**					
CRISPR 2	320994	323320	34	36	2,326
CRISPR 4	758381	758905	7	36	524
CRISPR 7	3165645	3165919	3	26	274
Possible CRISPR 1	212275	212374	1	32	99
Possible CRISPR 3	758039	758235	2	36	196
Possible CRISPR 5	1334949	1335043	1	26	94
Possible CRISPR 6	2395248	2395326	1	23	78
Possible CRISPR 8	3207039	3207149	1	29	110
***Flavobacterium columnare* ATCC 49512**					
CRISPR 1	391564	394476	43	36	2,912
CRISPR 2	1679967	1680530	8	36	563
Possible CRISPR 3	1994455	1994554	1	23	99

By comparison, the *F. columnare* 94-081 genome contains three CRISPR loci with 34, 7, and 3 spacers. DR lengths are 36, 36, and 26 bp, and the sequences are different for each CRISPR locus. The first CRISPR locus is located between CRISPR-associated genes *Cas*9 and *Cas*2. The second CRISPR locus is located between genes encoding two hypothetical proteins. The third CRISPR locus interrupts a gene encoding the cell envelope biogenesis protein *OmpA* (AWN65_RS13895). The *F. columnare* 94-081 genome has five possible CRISPRs-like structures with only two or three spacers in each (**Table 4**). Possible CRISPR 5 is located in a gene encoding a hypothetical protein (AWN65_RS05930). Possible CRISPR 3 is located near the second CRISPR locus, and the other possible CRISPRs are located between genes encoding hypothetical proteins. The strain 94-081 genome encodes *Cas*9 type II, *Cas*1 type II, and *Cas*2 type I-II-III proteins.

### Phage Sequences

The *F. columnare* ATCC 49512 genome carries three incomplete phage clusters in different locations on the genome. The lengths of the phage clusters are 6.3, 7, and 11.6 kb. Phage sequences were searched against *Flavobacterium* genomes using BLASTX, and 31 genes were determined (**Table 5**). Seven of these genes encode possible virulence factors in MvirDB.

**Table 5:** Phage elements in *F. columnare* ATCC 49512 and 94-081.

		Location	Locus tag	Product
*Flavobacterium columnare* strain 94-081	Region I	175781-176356	AWN65_RS00815	Guanylate kinase
		176392-176757	AWN65_RS00820	Four helix bundle protein
		176791-177372	AWN65_RS00825	Nicotinic acid mononucleotide adenylyltransferase
		177495-178397	AWN65_RS00830	*N*-Acetylmuramoyl-L-alanine amidase
		179325-180776	AWN65_RS00835	Nicotinate phosphoribosyltransferase
		180901-181632	AWN65_RS00840	Metallophosphatase
		181665-183095	AWN65_RS00845	ATPase
		183207-183656	AWN65_RS00850	Hypothetical protein
		183762-184319	AWN65_RS00855	RNA 2’-phosphotransferase
		184558-185193	AWN65_RS00860	hypothetical protein
		185330-185518	AWN65_RS00865	Hypothetical protein
		185635-186198	AWN65_RS00870	Hypothetical protein
		186284-187126	AWN65_RS00875	Crystallin J1
		187123-187659	AWN65_RS00880	Hypothetical protein
		187656-188486	AWN65_RS00885	Phosphoribosylpyrophosphate synthetase
		188909-189601	AWN65_RS00890	NUDIX hydrolase

*Flavobacterium columnare* ATCC 49512	Region I	1241010-1241513	FCOL_RS05470	Chromosome partitioning protein ParB
		1241510-1242790	FCOL_RS05475	Phosphoadenosine phosphosulfate sulfurtransferase
		1242787-1244013	FCOL_RS05310	Hypothetical protein
		1244047-1244736	FCOL_RS05485	Hypothetical protein
		1244733-1245056	FCOL_RS05490	Hypothetical protein
		1245075-1245617	FCOL_RS05495	hypothetical protein
		1245798-1246442	FCOL_RS05500	Hypothetical protein
		1246439-1246918	FCOL_RS05505	Peptidoglycan L-alanyl-D-glutamate endopeptidase
		1246890-1247372	FCOL_RS05510	Hypothetical protein
	
	Region II	2941840-2942271	FCOL_RS14160	Hypothetical protein
		2942993-2943172	FCOL_RS12865	Transposase
		2943259-2944173	FCOL_RS12870	Transposase
		2944446-2945636	FCOL_RS12875	Acyltransferase
		2945889-2946626	FCOL_RS12880	Capsular polysaccharide biosynthesis protein
		2946753-2947616	FCOL_RS12885	ABC transporter permease
		2947619-2948905	FCOL_RS12890	ABC transporter ATP-binding protein
	
	Region III	3022580-3023155	FCOL_RS13225	Guanylate kinase
		3023191-3023556	FCOL_RS13230	Four helix bundle protein
		3023645-3024157	FCOL_RS13235	Nicotinate-nicotinamide nucleotide adenylyltransferase
		3024280-3025182	FCOL_RS13240	*N*-Acetylmuramoyl-L-alanine amidase
		3025996-3027444	FCOL_RS13245	Nicotinate phosphoribosyltransferase
		3027503-3028006	FCOL_RS13250	Hypothetical protein
		3028052-3028456	FCOL_RS13255	Hypothetical protein
		3028541-3029266	FCOL_RS13260	Metallophosphatase
		3029263-3029805	FCOL_RS13265	RNA 2’-phosphotransferase
		3029802-3030464	FCOL_RS13270	Hypothetical protein
		3030527-3031030	FCOL_RS13275	RNase III inhibitor
		3031196-3031753	FCOL_RS13280	Hypothetical protein
		3031753-3032286	FCOL_RS13285	Hypothetical protein
		3032283-3033113	FCOL_RS13290	Phosphoribosylpyrophosphate synthetase
		3033491-3034183	FCOL_RS13295	NUDIX hydrolase

The *F. columnare* 94-081 genome contains one incomplete phage cluster of 13.8 kb with 16 genes, two of which have significant similarity with proteins in MvirDB. Eight of the phage genes were common in both *F. columnare* genomes. Phage elements in the *F. columnare* genomes are listed in **Table 5**.

### Genomic Islands and Insertion Elements

**FIGURE 4:**
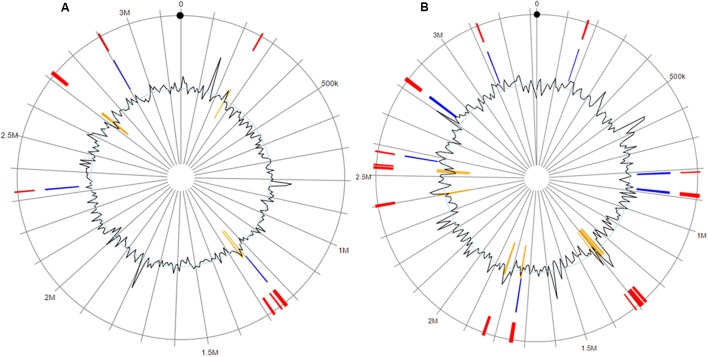
Genomic islands in the genomes of the *F. columnare* strain ATCC 49512 **(A)** and strain 94-081 **(B)**. Red: integrated prediction methods; orange: SIGI-HMM prediction methods; blue: IslandPath-DIMOB prediction method.

**FIGURE 5:**
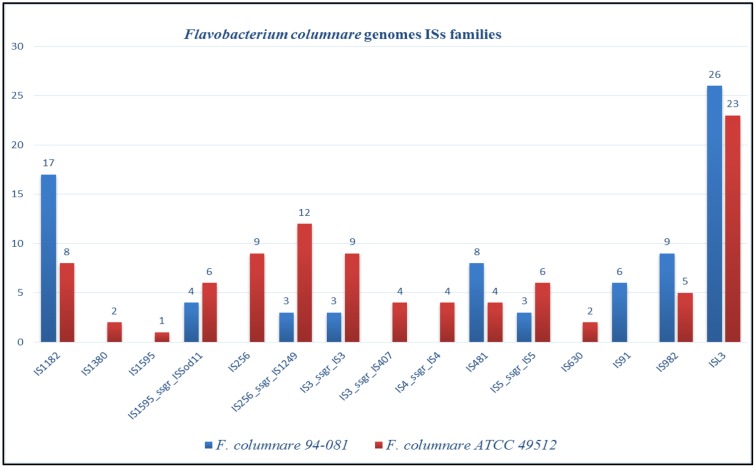
Insertion sequences (ISs) in the genomes of *F. columnare* strains ATCC 49512 and strain 94–081.

The *F. columnare* ATCC 49512 genome contains eight integrated GIs encoding 71 proteins, 50 of which are hypothetical. On the other hand, the *F. columnare* 94-081 genome has 15 integrated GIs encoding 162 proteins, 125 of which are hypothetical (**Figure 4**). Transposase, integrase, and DNA repair protein were encoded in GIs from both strains. Glycerol transferase, IS481 family transposase, and DNA-directed RNA polymerase sigma-70 factor were unique in GIs from strain ATCC 49512, whereas AAA family ATPase, ABC transporter ATP-binding protein, and flagellar motor protein MotB were unique to strain 94-081. The *F. columnare* ATCC 49512 genome contains 14 IS families with 95 interspersed protein-coding genes, while the *F. columnare* 94-081 genome has nine IS families with 79 interspersed predicted protein-coding genes (**Figure 5**).

### Regulatory Systems

The *F. columnare* strain ATCC 49512 genome encodes 32 predicted two-component system proteins, 58 transcription factor proteins, and 7 other DNA-binding proteins. The *F. columnare* strain 94-081 genome encodes 32 two-component system proteins, 74 transcription factor proteins, and 6 other DNA-binding proteins (**Table 6**). Although they have the same number of two-component systems, the distribution of histidine kinases (HK), response regulators (RR), and phosphotransferase proteins (PP) is different between the two strains. Strain 94-081 has more regulatory capacity than strain ATCC 49512 primarily due to the number of transcriptional regulator (TR) proteins.

**Table 6:** Regulatory systems in *F. columnare* ATCC 49512 and 94-081.

*F. columnare*	Predicted regulatory proteins							
	Two component systems			Transcription factors				Other DNA-binding proteins
	HK	RR	PP	TR	OCS	RR	SF	ODP
94-081	13	15	4	49	9	9	7	6
ATCC 49512	15	14	3	35	7	9	7	7

*HK, histidine kinases; RR, response regulators; PR, phosphotransferase proteins; TR, transcriptional regulators; OCS, one-component systems; SF, sigma factors; ODP, other DNA-binding proteins*.

Each of the two strains encodes unique regulatory proteins; strain ATCC 49512 encodes a unique transposase, cupin, PAS/PAC sensor signal transduction HK, and integration host factor subunit beta. Strain 94-081 encodes unique sensor HK, AAA family ATPase, ATP-binding protein, and HxIR family TR.

### Orthology Analysis

Orthology analysis by OrthoVenn showed that the *F. columnare* strain ATCC 49512 and strain 94-081 genomes contained 2,263 intersecting orthologous clusters. Strain ATCC 49512 has 290 unique orthologous clusters, which is composed of 274 single copy genes and 16 clusters with ≥2 paralogs. Strain 94-081 has 391 unique orthologous clusters with 370 single copy genes and 21 clusters with ≥2 paralogs. In its unique clusters, 28 of the *F. columnare* ATCC 49512 genes encode hypothetical proteins, and 18 encode transposases. Proteins unique tp strain ATCC 49512 included two Rhs element Vgr proteins, two peptidoglycan L-alanyl-D-glutamate endopeptidases, and one RHS repeat-associated core domain protein. In the *F. columnare* 94-081 genome, 90 of the total unique proteins were hypothetical, two were TRs, two were XRE family TRs, and two were endonucleases.

### Virulence Factors

Protein sequences of strains ATCC 49512 and 94-081 were searched against MvirDB. Results indicated that strain ATCC 49512 encoded a high number of proteins matching transposases in MvirDB, while strain 94-081 encoded a large number of proteins matching hypothetical proteins in the virulence database. MvirDB searches with a cutoff E-value of 10^-10^ indicated that *F. columnare* strain ATCC 49512 encodes 567 potential virulence proteins and *F. columnare* 94-081 encodes 592 potential virulence proteins (Supplementary Tables 1, 2). Proteins matches in the MvirDB database by both strains include 16S rRNA dimethyltransferase, 23S rRNA methyltransferase RlmB, an ABS transporter, a cold shock protein, gliding motility lipoproteins GldJ and GldK, molecular chaperones DnaJ, DnaK, GroEL, and HtpG, and secretion protein HlyD. In strain ATCC 49512, unique MvirDB matches include cell envelope biogenesis protein OmpA, multidrug ABC transporter permease/ATPase, multi-sensor hybrid HK, and sigma-54-dependent Fis family TR. Unique MvirDB matches from strain 94-081 include a hemolysin, λ family ATPase, Clp protease ClpC, an efflux transporter periplasmic adaptor subunit, flagellar motor protein MotB, and transcription factors.

### Mutational Analysis

**FIGURE 6:**
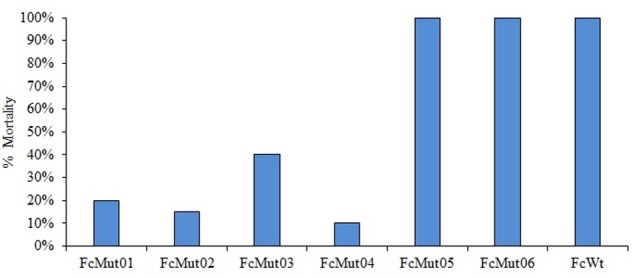
Mean percent mortalities resulting from experimental infection of channel catfish by *F. columnare* strain 94-081 and strain 94-081 Tn4351 insertion mutants (FcMut01: chalcone isomerase; FcMut02: hemolysin; FcMut03: anhydro-N-acetylmuramic acid kinase (AnmK); FcMut04: glycine cleavage system protein P (GcvP); FcMut05: transcriptional regulator TetR, FcMut06: peptidoglycan-binding protein LysM.

Using erythromycin at 10 μg/ml, our conjugation yielded six *F. columnare* strain 94-081 Tn4351 mutants (**Table 7**). All six colonies contained transposon insertions; no background non-mutant colonies occurred. Wild-type strain 94-081 caused 100% mortality in catfish fingerlings, while FcMut01 [*F. columnare* Tn4351 chalcone isomerase (CHI) insertion mutant] and FcMut02 (hemolysin mutant) had averages of 20 and 15% mortalities, respectively. Additionally, FcMut03 (anhydro-*N*-acetylmuramic acid kinase mutant) had an average of 40% mortalities, and FcMut04 (glycine dehydrogenase mutant) had an average of 10% mortalities. FcMut05 and FcMut06 had similar mortalities to the wild-type strain (**Figure 6**).

**Table 7:** Tn4351 insertion mutants of *F. columnare* 94-081.

Mutants	Transposon end	Locus tag	Gene ID
FcMut01	F	AWN65_RS03755	Chalcone isomerase
FcMut02	R	AWN65_RS11020	Hemolysin
FcMut03	F	AWN65_RS03750	Anhydro-*N*-acetylmuramic acid kinase (AnmK)
FcMut04	R	AWN65_RS11110	Glycine cleavage system protein P (GcvP)
FcMut05	R	AWN65_RS03940	Transcriptional regulator TetR
FcMut06	R	AWN65_RS04445	Peptidoglycan-binding protein LysM

## Discussion

*F. columnare* strains are divided into three genomovar groups based on their colony morphology and genetic heterogeneity (Triyanto and Wakabayash, 1999). The purpose of this study was to compare the genomes of *F. columnare* strain ATCC 49512 (genomovar I) and *F. columnare* strain 94-081 (genomovar II) (Tekedar et al., 2012; Kumru et al., 2016). Evaluation of both strains in catfish by immersion challenge showed that *F. columnare* strain ATCC 49512 was not virulent in catfish, while *F. columnare* strain 94-081 was highly virulent (Soto et al., 2008). Among the three genomovar groups, genomovar II contains the most *F. columnare* strains that are virulent to catfish (Triyanto and Wakabayash, 1999; Arias et al., 2004; Darwish and Ismaiel, 2005; Olivares-Fuster et al., 2007; Shoemaker et al., 2008; Bullard et al., 2013).

*F. columnare* strain ATCC 49512 and strain 94-081 are the first complete genomes in their corresponding genomovars; the genomovar III group does not have a representative complete genome yet. *F. columnare* genomes are bigger than *F. psychrophilum* (2,860,382 bp) but smaller than *F. branchiophilum* (3,559,884 bp) and *F. johnsoniae* (6,096,872 bp). In *F. columnare*, the strain 94-081 genome (3,331,600 bp) is 169 kb bigger than the strain ATCC 49512 genome (3,162,432 bp). Furthermore, strain 94-081 has 147 more predicted protein-coding genes and has one less ribosomal RNA operon compared to strain ATCC 49512 (**Table 2**). RAST annotation showed that both genomovars share similar subsystems (**Figure 1**).

Genome alignment by MAUVE showed local synteny but very different overall alignments. ANI between the two strains (90.71%) and DDH determination (42.60%) indicate that the two genomovars could be considered different species. The accepted ANI cutoff to define two strains as the same species is greater than 95% (Goris et al., 2007). Similarly, the recommended DDH cutoff to define strains as a species is >70% (Meier-Kolthoff et al., 2013, 2014). Phylogenetic analysis of all the available complete genomes in the *Flavobacterium* genus shows that *F. columnare* genomovar I and genomovar II strains cluster separately, but they are more closely related to each other than other species (**Figure 3**). *F. indicum* GPTSA100-9 and *F. psychrophilum* are the closest species to *F. columnare*. Based on these criteria, our results suggest these two strains could be considered different species.

Bacterial secretion systems are necessary for bacterial growth, virulence, and competition (Green and Mecsas, 2016), and there are several types of bacterial secretion systems (Gerlach and Hensel, 2007; Sato et al., 2010; Dalbey and Kuhn, 2012; Xu and Luo, 2013). The *F. columnare* strain ATCC 49512 and strain 94-081 genomes both have complete T1SS, T6SS^iii^, and T9SS as well as other partial secretion system elements (**Table 3**). Proteases, lipases, hemolysins, and leukotoxins are secreted by T1SS in pathogens (Akatsuka et al., 1995; Dirix et al., 2004; Bleves et al., 2010; Kanonenberg et al., 2013). Strain ATCC 49512 encodes more T1SS structural proteins than strain 94-081.

Type VI secretion system has three subtypes, including a new subtype unique to the *Bacteroidetes* first described as T6SS^iii^ in *F. johnsoniae* (Russell et al., 2014). T6SS contributes to virulence of Gram-negative bacteria by transferring toxins to host cells or preventing the growth of other bacteria (Zoued et al., 2014; Aubert et al., 2015; Costa et al., 2015). Although both strains have a T6SS^iii^, the number of TssD (Hcp) and TssI (VgrG) secretion proteins encoded in the two strains is different (**Table 3**). TssD (Hcp) and TssI (VgrG) are bacteriophage-related proteins interacting with non-overlapping sets of effectors. While Hcp is ring-shaped and interacts with effectors within its pore, VgrG is a phage tail spike-like protein that interacts with effectors via conserved adaptor domains. Strain 94-081 has more VgrG and Hcp proteins than strain ATCC 49512, which could contribute to genomovar II pathogenicity in catfish.

Type IX secretion system or PorSS was identified first in *Porphyromonas gingivalis* and *F. johnsoniae*; this secretion system is also common in the *Bacteroidetes* phylum (McBride, 2001; Sato et al., 2010; McBride and Zhu, 2013; Kharade and McBride, 2015). In the *Bacteroidetes* phylum, T9SS is a protein carriage system for bacteria, and it is important for surface motility protein secretion and adhesion in *F. johnsoniae* (Shrivastava et al., 2013). Both genomovar strains have similar T9SS and motility proteins.

In prokaryotes, CRISPR systems are the “memory” component of a genetic adaptive immune system (Touchon et al., 2011). Cas (CRISPR-associated family genes) system provides the mechanism of prokaryotic resistance to foreign DNA (Mojica et al., 2005; Pourcel et al., 2005; Makarova et al., 2006). CRISPR-Cas proteins may control gene expression and regulate bacterial virulence (Hatoum-Aslan and Marraffini, 2014; Westra et al., 2014). Generally, up to 16 CRISPR clusters have been identified in prokaryotic genomes with the same or different numbers of DRs, which can vary in size between 24 to 47 bp (Mojica et al., 1995; Jansen et al., 2002; Bolotin et al., 2005; Barrangou et al., 2007). Spacer sequences are usually between 0.6 and 2.5X DR size, and spacers are the CRISPR immunity targets (Stern et al., 2010; Brodt et al., 2011). Some CRISPR systems are classified as “possible” because their DRs are not 100% identical (Grissa et al., 2007). Strain 94-081 has eight CRISPR loci (including five “possible”), and strain ATCC 49512 has three (one “possible”). Although strain 94-081 has more CRISPR loci than strain ATCC 49512, the total number of DR in CRISPR is very similar between to the two strains. Strain 94-081 has more CRISPR-Cas proteins, but the significance of this in bacterial pathogenesis is unknown.

Phages and prophages (lysogenic bacteriophage inserted into bacterial chromosomes and plasmids) contribute to bacterial environmental adaptation, antibiotic resistance, or pathogenicity. Bacteriophages can carry bacterial virulence genes, and chromosomal integration can inactivate bacterial genes (Casjens, 2003; Chibani-Chennoufi et al., 2004; Coates and Hu, 2007; Zhou et al., 2011; Arndt et al., 2016). Bacteriophages can be inactive, and some are activated in host cells to express virulence functions (Casjens, 2003; Coates and Hu, 2007; Zhou et al., 2011; Arndt et al., 2016). Strain ATCC 49512 has more prophage regions and proteins than strain 94-081, which may allow some unique environmental adaptations.

Genomic islands are clustered genes in prokaryotic genomes, and they play a major role in microbial genome evolution. They are commonly considered to originate from horizontal gene transfer and can encompass large genomic regions. Often they encode adhesion proteins, toxins, T3SSs, iron uptake proteins, antibiotic resistance proteins, or virulence factors (Hacker and Carniel, 2001; Whittle et al., 2002; Dobrindt et al., 2004; Gal-Mor and Finlay, 2006; Langille et al., 2010). IslandViewer3 integrates three of the most accurate GI prediction methods, IslandPick, IslandPath-DIMOB, and SIGI-HMM, which utilize different prediction methods (Hsiao et al., 2003; Waack et al., 2006; Langille et al., 2008; Langille and Brinkman, 2009; Dhillon et al., 2015). Strain 94-081 has more GIs, most of which encode hypothetical proteins. Twenty-four proteins encoded in GIs from strain 94-081 have significant matches with virulence proteins in MvirDB, while 15 of the GI proteins in strain ATCC 49512 have matches with proteins in MvirDB.

Insertion sequences are self-directed mobile genetic elements that contribute to horizontal gene transfer and genome organization (Varani et al., 2011). IS elements range from 0.7 to 3.5 kb in size, but they are usually less than 2.5 kb (Mahillon and Chandler, 1998; Siguier et al., 2006). IS elements appear to contribute to evolution of pathogenic bacterial genomes (Moran and Plague, 2004; Song et al., 2010; Schmitz-Esser et al., 2011; Varani et al., 2011), and movement of IS in the genome can contribute to antibiotic resistance (Tan, 1999; Wagner, 2006). Strain ATCC 49512 has more IS families and genes than strain 94-081, possibly indicating increased horizontal gene exchange has occurred.

Transcriptional regulation by transcription factors and two-component system proteins is important for microbial adaptation (Barakat et al., 2013). Two-component signal transduction systems (TCS) consist of HKs and a RR. These systems modulate gene expression in response to changing environmental conditions, including pathogenic events such as invasion of host cells, biofilm formation, and resistance to antibiotics (Ernst et al., 1999; Laub, 2011; Dsouza et al., 2014; Kadowaki et al., 2016). Other types of transcriptional regulatory factors include sigma factors (SF) and one component systems (OCS) (Osterberg et al., 2011; Barakat et al., 2013). The *F. columnare* 94-081 genome contains 18 more transcription factor proteins and two component system elements than the *F. columnare* ATCC 49512 genome, suggesting a more complex regulatory network.

Orthologous genes (clusters of genes derived from a common ancestor) (Fitch, 1970) are useful for comparative analysis of genome functional pathways. Orthologous cluster comparison can also be helpful for phylogenetic analysis (Henikoff et al., 1997; Mushegian et al., 1998; Wang et al., 2015). Interestingly, even though ANI and DDH analysis indicate strains ATCC 49512 and 94-081 could be considered different species, and genome alignment indicates extensive genomic rearrangements, strain ATCC 49512 and strain 94-081 have a large core of orthologous clusters (2,263 total). This indicates that despite their genomic differences, ATCC 49512 and 94-081 are functionally similar. Therefore, phenotypic differentiation of genomovar I and genomovar II strains may not be feasible. Interestingly, the relatively low number of unique proteins to genomovar II strains mediate a large difference in virulence in catfish; further study of these proteins is warranted. Eleven of the unique proteins from strain 94-081 are encoded in GIs, and three of the unique proteins from strain ATCC 49512 are encoded by GIs, suggesting horizontal gene transfer as the source.

Identification of virulence factors is important for understanding bacterial pathogenesis and host/pathogen interactions (Wu et al., 2008). Comparison of the strains’ predicted proteomes against MvirDB indicated a similar number of potential virulence genes (567 for strain ATCC 49512 and 592 for strain 94-081). GIs frequently carry virulence genes, and many of the virulence genes unique to genomovar II are in GIs (Zhou et al., 2007). Some of the unique predicted virulence proteins from genomovar II are involved in secretion systems and regulatory proteins.

A random transposon mutagenesis method was adapted to *F. columnare* genomovar II strain 94-081. Previously, transfer of plasmid pCP29 by conjugation into strain 94-081 was reported (Staroscik et al., 2008), but to our knowledge the current study is the first report of transposon mutagenesis in this strain. Similar to previously reported transposon mutagenesis using Tn4351 in *F. columnare* (Staroscik et al., 2008), the efficiency of mutagenesis was low (a total of six mutants were obtained from a single conjugation), so refinement of this method is warranted. We used a higher concentration of erythromycin (10 μg/ml) than previously reported (Staroscik et al., 2008), which eliminated background colonies. Virulence of the mutants was compared to parent strain 94-081 using an established model with channel catfish. Three of the mutants caused <20% mortalities (compared to 100% mortalities for wild-type strain). One of these had an insertion in a gene encoding CHI, which is an enzyme involved in flavone/flavanone degradation in bacteria. The human intestinal anaerobic bacterium *Eubacterium ramulus* expresses this enzyme (Braune et al., 2016), but it has not been implicated in virulence. Another mutation that caused attenuation was in a gene encoding a hemolysin, which can be an important virulence factor (Rowe and Welch, 1994; Zhang and Austin, 2000). In *F. psychrophilum*, hemolytic activity is contact dependent and mediated by a thermolabile enzyme (Hogfors-Ronnholm and Wiklund, 2010). The third mutant was in glycine cleavage system protein P (*gcvP*). The glycine cleavage system was linked to virulence in the fish pathogen *Edwardsiella ictaluri*, where the enzyme was shown to be involved in neutrophil and serum resistance (Karsi et al., 2009; Dahal et al., 2013).

Mutation of the gene encoding anhydro-*N*-acetylmuramic acid kinase (*anmK*) in 94-081 also caused attenuation, but mean percent mortalities were higher (>40%). In *Francisella*, the *anmK* gene is necessary for full virulence, but it is not required for intracellular growth (Ludu et al., 2008). Two *F. columnare* 94-081 mutants were not attenuated. One of the mutations was in a TetR family TR, which typically function as transcriptional repressors and can regulate antibiotic resistance, catabolic pathways, quorum sensing, and virulence of other pathogenic bacteria (Krushkal et al., 2011; Cuthbertson and Nodwell, 2013). The 94-081 *lysM* mutant was also not attenuated; LysM is a peptidoglycan-binding protein that is widely distributed in prokaryotes. In some bacteria, LysM mediates attachment to extracellular matrix (Downer et al., 2002).

In summary, comparison of the genomes from *F. columnare* genomovar I strain ATCC 49512 and genomovar II strain 94-081 provided some evidence that the two genomovars could be considered separate species (based on ANI and DDH analysis). However, orthology analysis revealed a largely conserved core genome, indicating that phenotypically the two genomovars are very similar. In particular, core metabolic functions are similar between the two genomovars. Both genomovars have CRISPR-Cas systems and evidence of horizontal gene acquisition, and despite local syntenic regions, a large number of genomic rearrangements are present between the two. For the first time, we report transposon mutagenesis of a genomovar II strain; this tool along with the genome sequence and an established infection model in catfish will enable future elucidation of mechanisms of pathogenesis in this important fish pathogen.

## Author Contributions

Designed the experiments: SK, HT, NG, GW, ML, and AK. Conducted comparative genomics analysis: SK and HT. Transposon mutagenesis and fish challenge were performed by NG. The manuscript was written by SK, HT, ML, and AK. All authors read and accepted the final manuscript.

## Conflict of Interest Statement

The authors declare that the research was conducted in the absence of any commercial or financial relationships that could be construed as a potential conflict of interest.

## Footnotes

1 http://www.pathogenomics.sfu.ca/islandviewer

2 http://issaga.biotoul.fr/ISsaga2/public/issaga_public.php

3 http://www.p2rp.org/

4 http://www.bioinfogenome.net/OrthoVenn/

5 http://mvirdb.llnl.gov/
